# The Carbon monoxide releasing molecule ALF-186 mediates anti-inflammatory and neuroprotective effects via the soluble guanylate cyclase ß1 in rats’ retinal ganglion cells after ischemia and reperfusion injury

**DOI:** 10.1186/s12974-017-0905-7

**Published:** 2017-06-27

**Authors:** Felix Ulbrich, Claus Hagmann, Hartmut Buerkle, Carlos C. Romao, Nils Schallner, Ulrich Goebel, Julia Biermann

**Affiliations:** 1 Department of Anesthesiology and Critical Care, Medical Center, University of Freiburg, Faculty of Medicine, University of Freiburg, Hugstetter Strasse 55, D-79106 Freiburg, Germany; 2 Eye Center, Medical Center – University of Freiburg, Faculty of Medicine, University of Freiburg, Freiburg, Germany; 3 Instituto de Tecnologia Química e Biológica-António Xavier, Universidade Nova de Lisboa, Oeiras, Portugal; 4 Alfama Ltd., Instituto de Biologia Experimental e Tecnológica, IBET, Oeiras, Portugal

**Keywords:** Ischemia/reperfusion injury, Carbon monoxide, Neuro-inflammation, Neuroprotection, sGC-ß_1_, Heat shock proteins

## Abstract

**Background:**

The endogenously produced gaseous molecule carbon monoxide is able to promote organ protection after ischemia-reperfusion injuries (IRI). The impact of carbon monoxide releasing molecules (CORM) regarding inflammation in neuronal tissues has not been studied in detail. In this investigation, we aimed to analyze the effects of the CORM ALF-186 on neuro-inflammation and hypothesized that the soluble guanylate cyclase (sGC) is playing a decisive role.

**Methods:**

Retinal ischemia-reperfusion injury was performed for 60 min in Sprague-Dawley rats. Thereafter, the CORM ALF-186 (10 mg/kg) in the presence or absence of the sGC inhibitor ODQ was injected via a tail vein. Retinal tissue was harvested 24 h later to analyze mRNA or protein expression of sGC-β1 subunit, transcription factors NF-κB and CREB, the inflammatory cytokines TNF-α and IL-6, as well as the heat shock proteins (HSP) HSP-70 and HSP-90. Immunohistochemistry was performed on frozen sections of the retina. The overall neuroprotective effect of ALF-186 was assessed by counting fluorogold-pre-labeled retinal ganglion cells (RGC) 7 days after IRI.

**Results:**

Ischemia-reperfusion mediated loss of vital RGC was attenuated by the administration of ALF-186 after injury. ALF-186 treatment after IRI induced sGC-ß_1_ leading to a decreased NF-κB and CREB phosphorylation. Consecutively, ALF-186 mitigated IRI induced TNF-α and IL-6 expression in the retina and in the rats’ serum. Moreover, ALF-186 attenuated heat shock protein 70 (Hsp-70) while increasing Hsp-90. The sGC-inhibitor ODQ attenuated the anti-inflammatory effects of ALF-186 and increased retinal loss of ganglion cells. These results were confirmed by immunohistochemistry.

**Conclusion:**

The CORM ALF-186 protected RGC from IRI induced loss. Furthermore, ALF-186 reduced IRI mediated neuroinflammation in the retina and in the serum by activating sGC. Inhibition of sGC stopped the beneficial and protective effects of ALF-186. ALF-186 may present a promising therapeutic alternative in treating inflammation after neuronal IRI.

## Background

Exogenously administered carbon monoxide ameliorates injuries of various organs like the heart, lung, kidney, and liver. Efficiency of inhaled carbon monoxide [1] and carbon monoxide releasing molecule (CORM), given before or after injury, has been proven in many animal models using ischemia-reperfusion injury (IRI), transplantation, or sepsis [2–8]. Interfering with physiological intracellular processes CO affects among others inflammation and apoptosis leading to reduced cell death [9, 10]. However, CO’s impact on neuronal tissue is less well investigated and the underlying mechanisms have not been fully understood.

Ischemic injury in neurons after stroke or traumatic brain injury is one of the leading causes of mortality. Due to high sensitivity of insufficient blood flow and oxygen supply of neurons as well as limited regeneration ability, ischemic injury is frequently associated with poor neurological outcome [11–13]. Therefore, restoration and maintenance of cerebral circulation must be the primary goal of treatment.

Since there is currently no reliable medical opportunity for mitigating neuronal injury, CO might be a possible agent for this approach.

Querioga et al. showed in their study that inhaled carbon monoxide as a preconditioning agent provides neuroprotection following perinatal hypoxia-ischemia [14]. Given before injury, carbon monoxide exerts its protective effect, at least in part, via the soluble guanylate cyclase (sGC) [1]. As an endogenous receptor for nitric oxide sGC is activated by CO-binding on its heme groups and catalyzes cGMP activation [15].

We hypothesize that the CORM ALF-186 would reduce neuroinflammation after retinal IRI and mediate neuroprotective effects via the sGC pathway.

## Methods

### Animals

Adult male and female Sprague-Dawley rats (1:1, 280–350 g bodyweight, Charles River, Sulzfeld, Germany) were used in these experiments. Animals were fed with a standard diet ad libitum, being kept on a 12 h light/12 h dark cycle. All procedures involving animals concurred with the statement of The Association for Research in Vision and Ophthalmology for the use of animals in research in accordance with the ARRIVE guidelines and were approved a priori by the Committee of Animal Care of the University of Freiburg (Permit No: 35-9185.81/G-11/81). All types of surgery and manipulations were performed under general anesthesia with isoflurane/O_2_ for retrograde labeling with fluorogold (FG) or with a mixture of intraperitoneally administered ketamine 50 mg/kg (Ceva-Sanofi, Duesseldorf, Germany) and xylazine 2 mg/kg (Ceva-Sanofi) for the ischemia-reperfusion experiment. Body temperature was maintained at 37 ± 0.5 °C with a heating pad. After surgery, buprenorphine (Temgesic® 0.5 mg/kg; Essex Pharma, Solingen, Germany) was applied intraperitoneally to prevent pain. During recovery from anesthesia, the animals were placed in separate cages. The number of animals used for RGC quantification and molecular analysis was *n* = 8 per group. For analysis of mRNA- and protein-expression, retinal tissue was harvested at *t* = 24 h after reperfusion.

### Retrograde labeling of RGC

Sprague Dawley rats were anesthetized, placed in a stereotactic apparatus (Stoelting, Kiel, Germany) and retrograde RGC-labeling was done as described previously [16], briefly summarized as follows. The skin overlying the skull was cut open und retracted. The lambda and bregma sutures served as landmarks for drilling three holes on each site of the bregma sutures. A total amount of 7.8 μl fluorogold (FG) (Fluorochrome, Denver, CO, USA) dissolved in DMSO/PBS was injected into both superior colliculi through the drilling holes. To ensure adequate retrograde transport of FG into the RGC’s perikarya, further experimental interventions were done 7 days after retrograde labeling.

### Retinal ischemia/reperfusion injury and treatment with ALF-186

Following randomization, rats were sedated and the anterior chamber of the left eye was cannulated with a 30-gauge needle connected to a reservoir containing 0.9% NaCl. Intraocular pressure was increased to 120 mm Hg for 60 min and ocular ischemia was confirmed microscopically by interruption of the retinal circulation. Reperfusion was initiated by removing the needle tip promptly. Rats without immediate recovery of retinal perfusion at the end of the ischemic period or those with lens injuries were excluded from the study, since the latter prevents RGC death and promotes axonal regeneration. To evaluate a neuroprotective effect of carbon monoxide released from ALF-186, animals were randomly assigned to receive treatment either with ALF-186 (10 mg/kg body weight i.v., dissolved in water) or water (vehicle control) alone. Both were injected immediately after IRI or with a delay of 3 h to IRI. Inactivated ALF-186 (iALF-186) was used 24 h after dissolving to exclude crucial effects of the molybdenum heavy metal backbone. Inactivated ALF-186 (iALF) was produced by dissolving ALF-186 in PBS and allowing the liquid solution to stand overnight open to room air and accessible for light. In this time, ALF-186 was able to release CO from its chemical structure, leaving only the heavy-metal backbone (i.e. molybdenum) inside the solution. Prior to the experiments where iALF was used as a negative control, the solution was bubbled with nitrogen, to remove any possible CO-molecules that might have been left in the solution. After that, ALF-186 rested for another hour.

In further experiments, rats received 60 min prior to IRI the sGC inhibitor ODQ (2.5 mg/kg bodyweight i.v., Sigma-Aldrich, Taufkirchen, Germany, #O2626).

### RGC quantification

Animals were sacrificed by CO2-inhalation 7 days after ischemia. Retinal tissue was immediately harvested, placed in ice-cold Hank’s balanced salt solution and further processed for whole mount preparation. Retinae were carefully placed on a nitrocellulose membrane with the ganglion cell layer (GCL) on top. After removing the vitreous body, retinae were fixed in 4% paraformaldehyde for 1 h and then embedded in mounting media (Vectashield; Axxora, Loerrach, Germany). The densities of FG-positive RGC were determined in blinded fashion using a fluorescence microscope (AxioImager; Carl Zeiss, Jena, Germany) and the appropriate bandpass emission filter (FG: excitation/emission, 331/418 nm), as previously described [16]. Briefly, we photographed 3 standard rectangular areas (0.200 × 0.200 mm = 0.04 mm^2^) at 1, 2, and 3 mm from the optic disc in the central regions of each retinal quadrant. Hence, we evaluated an area of 0.48 mm^2^ per retina. To calculate the average RGC density in cells/mm2, we multiplied the number of analyzed cells/0.04 mm^2^ with 25. Secondary fluorogold stained activated microglia cells (AMC) after RGC phagocytosis were identified by morphologic criteria and excluded from calculation. All data are presented as mean RGC densities [cells/mm^2^] ± SD.

### Immunohistochemical staining

Eyes were enucleated 24 h after intervention and immediately placed in 4% paraformaldehyde overnight at 4 °C. After washing in Dulbecco’s phosphate buffered saline (D-PBS) before and after postfixation in 30% sucrose for 5–6 h at room temperature, eyes were embedded in compound (Tissue-Tek; Sakura-Finetek, Torrance, CA, USA) and frozen in liquid nitrogen. Frozen serial sections (10 μm) were cut through the middle third of the eye and collected on gelatinized slides. Immunolabeling was performed according to standardized protocols with primary antibodies against soluble Guanylate Cyclase ß_1_ (#160897; rabbit, dilution 1:1500; Cayman Chemical, Ann Arbor, Michigan, USA), TNF-α and Hsp-90 (#ab6671 and #ab19021; rabbit, dilution 1:200; both Abcam, Cambridge, UK), IL-6 (#LS-C70904/65996; rabbit, dilution 1:600; Biozol, Eching, Germany) and Brn-3 (#sc-390078; rabbit, dilution 1:200; Santa Cruz Biotechnology, Dallas, TX, USA). Primary antibodies were then conjugated with their corresponding secondary antibody (red fluorescence: #211-605-109, Alexa Fluor 647, mouse anti-rabbit, dilution 1:1000; green fluorescence: #705-225-147, Cyanine Cy^TM^2, donkey anti-goat, dilution 1:1000; #111-225-045, Cyanine Cy^TM^2, goat anti-rabbit, dilution 1:1000; all Jackson ImmunoResearch Europe, Newmarket, UK). The nuclei of cells in the retina were stained with 4′,6-diamino-2-phenylindole dihydrochloride hydrate (DAPI, Sigma, Taufkirchen, Germany) added to the embedding medium (Mowiol; Calbiochem, San Diego, CA, USA). Slides were examined under a fluorescence microscope (Axiophot; Carl Zeiss, Jena, Germany).

Quantification and histogram analysis of immunostained images was performed using the ImageJ open source software (ImageJ, Version 2.00-rc-44/1.50e, open source image processing software).

### Western Blot analysis

24 h after ischemia retinal tissue for analysis of protein expression was harvested. Total protein from ¾ of retina was extracted and processed for Western Blot as described previously. The membranes were blocked with 5% skim milk in Tween20/PBS and incubated in the recommended dilution of protein specific antibody (p-NF-κB #3033, TNF-α #3707S, Cell Signaling Technology, Danvers, MA, USA, Hsp-70 #ab31010, Hsp-90 #ab13492, IL-6 #ab25107, Abcam, Cambridge, UK, sGC-β_1_ #160897 Cayman Chemical, Ann Arbor, Michigan, USA) overnight at 4 °C. After incubation with a horseradish peroxidase-conjugated anti-rabbit secondary antibody (GE Healthcare, Freiburg, Germany), proteins were visualized using the ECL plus Chemiluminescence Kit (GE Healthcare). For normalization, blots were re-probed with NF-κB (#8242, Cell Signaling) and ß-Actin (#4967S, Cell Signaling). Relative changes in protein expression in IR injured retinae either with injection of ALF-186 or PBS were calculated in relation to the corresponding non-ischemic retinae.

Densitometric analysis of individual phosphorylation was performed using the Image-J open source software (ImageJ, Version 2.00-rc-44/1.50e, open source image processing software) comparing the relative changes in protein expression in IR injured retinae with each intervention and calculated in relation to the corresponding non-ischemic retinae.

### Real-time polymerase chain reaction (RT-PCR)

From retinal tissue harvested 24 h after ischemia, total RNA from ¼ of retina was extracted using a column-purification based kit (RNeasy Micro Kit, Qiagen, Hilden, Germany) according to the manufacturer’s instructions. Reverse transcription was performed with 50 ng of total RNA using random primers (High Capacity cDNA Reverse Transcription Kit, Applied Biosystems, Darmstadt, Germany). Real-time polymerase chain reactions (RT-PCR) were done with TaqMan® probe-based detection kit (TaqMan® PCR universal mastermix, Applied Biosystems, Darmstadt, Germany). Following primers were used: NF-κB #Rn01399583_m1, CREB #Rn02532082_g1, TNF-α #Rn01525859_g1, IL-6 #Rn01410330_m1, Hsp-70 #Rn04224718_u1, Hsp-90 #RN00822023_g1 (all from Applied Biosystems, Darmstadt, Germany). The PCR assays were then performed on a RT-PCR System (ABI Prism 7000, Applied Biosystems, Darmstadt, Germany) with the following cycling conditions: 95 °C for 10 min, 40 cycles of 95 °C for 10 s and 60 °C for 1 min. Reaction specificity was confirmed by running appropriate negative controls. Cycle threshold (CT) values for each gene of interest were normalized to the corresponding CT values for GAPDH (ΔCT). Relative gene expression in IR injured retinal tissue either with injection of ALF-186 or PBS was calculated in relation to the corresponding gene expression in the non-injured retinal tissue of each individual animal (ΔΔCT).

### Statistical analysis

Data was analyzed using a computerized statistical program (SigmaPlot Version 11.0, Systat Software Inc., San Jose, CA, USA). The results are presented as means (±SD) after normal distribution of data had been verified. One-way ANOVA for repeated measurements was used for between-group comparisons with post hoc Holm-Sidak test and Kruskal Wallis test for data with lack of normal distribution. *P* < 0.05 was considered statistically significant.

## Results

### ALF-186 treatment protects RGC against ischemia reperfusion injury (IRI)

**Fig. 1:**
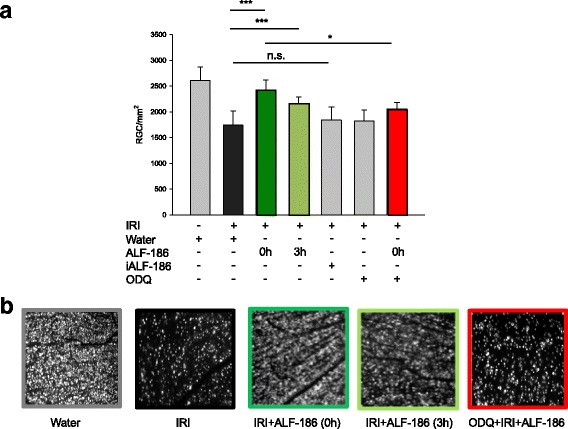
Influence of ALF-186 treatment on vital retinal ganglion cell count after ischemia reperfusion injury (IRI). **a** Quantification of retinal ganglion cell density (cells/mm^2^, data are mean ± SD, *n* = 8, IRI vs. IRI + ALF-186 0 h and 3 h, *** = *p* < 0.001 and IRI + ALF-186 0 h vs. IRI + ALF-186 + ODQ, * = *p* < 0.05; n.s. = not significant). **b** Representative flat mount images (*n* = 8) of fluorogold-labeled retinal ganglion cells 7 days after IRI, immediate and delayed (3 h) ALF-186 treatment, inactivated ALF-186 treatment and SGC inhibition with ODQ

Retinal IRI decreased the density of retinal ganglion cells by approximately 40% (Fig. 1, Col. 1 and 2; IRI 1808 ± 262 vs. untreated 2503 ± 295, *** = *p* < 0.001). Immediate (0 h) ALF-186 treatment almost completely neutralized this effect (untreated 2503 ± 295 vs. IRI + ALF-186 0 h 2499 ± 377). Even delayed ALF-186 application (3 h) reduced RGC loss significantly (IRI 1808 ± 262 vs. IRI ± ALF-186 3 h 2202 ± 239, *** = *p* < 0.001). In contrast, administration of inactivated ALF-186 did not influence RGC survival compared to untreated animals. The sGC inhibitor ODQ was able to partly inactivate the neuroprotective effect of ALF-186 (IRI + ALF-186 2499 ± 377 vs. ODQ + IRI + ALF-186 2126 ± 1680, * = *p* < 0.05).

### ALF-186 stimulates sGC-β_1_ protein expression

**Fig. 2:**
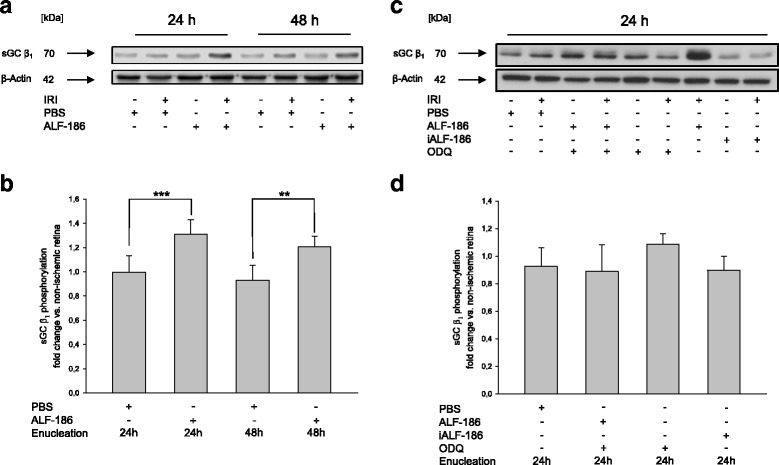
Effect of ALF-186 treatment on the expression of soluble guanylyl cyclase (sGC) β_1_. **a** Representative western blot image (*n* = 8) showing the increase of sGC β_1_ compared to β-Actin after immediate ALF-186 postconditioning. Enucleation was performed either 24 or 48 h after IRI. **b** Densitometric analysis of *n* = 8 western blots for sGC β_1_ after ALF-186 (data are mean ± SD; IRI vs. IRI + ALF-186 24 h, *** = *p* < 0.001 and IRI vs. IRI + ALF-186 48 h, ** = *p* < 0.01). **c** Representative western blot image and densitometric analysis of *n* = 8 Western Blots for sGC β_1_ compared to β-Actin after ODQ inhibition and ALF-186, ODQ inhibition alone and ODQ-inhibition and iALF-186. Enucleation was performed 24 h after IRI. **d** Densitometric analysis of *n* = 8 Western Blots for sGC β_1_ after ALF-186 in the presence of ODQ

**Fig. 3:**
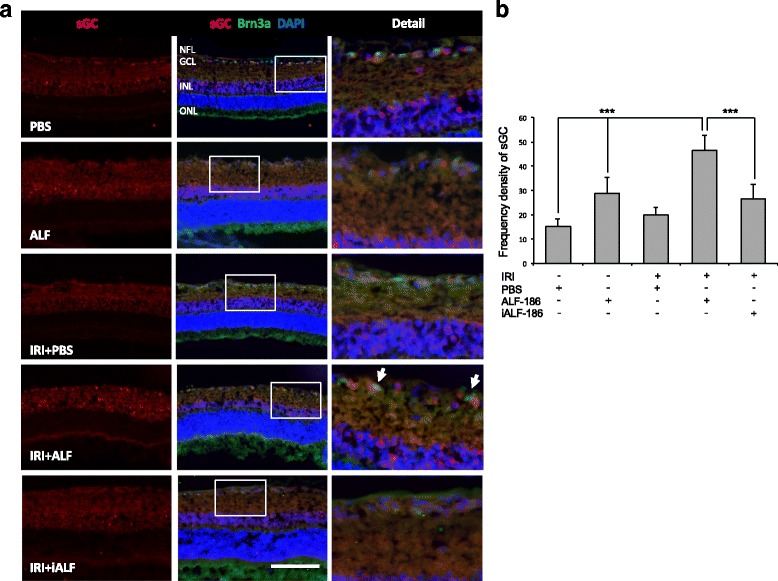
ALF-186 treatment increases sGC expression in retinal ganglion cells due to IRI **a** sGC and Brn3a/DAPI co-expression in the retina after unilateral IRI in the presence and abscence of ALF-186/iALF-186 or PBS. Cross-sections of the retinae 24 h after unilateral IRI. In controls with and without IRI sGC was only positive as a baseline expression. ALF-186 vehicle group and IRI + ALF-186 treatment resulted in an increased expression of sGC, mostly in the inner nuclear layer and the retinal ganglion cells as co-staining with Brn3a demonstrates (*right column*, *forth image*, *arrows*). Inactivated ALF-186 did not show any significant effects concerning sGC expression compared to control groups. (Scale bar, 50 μm; Abbreviations: NFL = nerve fiber layer, GCL = ganglion cell layer, INL = inner nuclear layer and ONL = outer nuclear layer). **b** Frequency density of sGC protein expression in the groups as quantified by histogram analysis (*** = *p* < 0.001 PBS vs. ALF-186 amd IRI + ALF-186 and IRI + ALF-186 vs. IRI + iALF-186)

In the following experiment, we determined sGC-β_1_ protein expression after IRI and ALF-186 treatment. ALF-186 increased sGC-β_1_ protein expression significantly. Concerning the time point of enucleation, major effect was detectable after 24 h. Performing enucleation 48 h after IRI the effect on sGC-β_1_ was reduced. (Fig. 2a, b; *** = *p* < 0.001; ** = *p* < 0.01). To confirm ALF’s mechanism of action on sGCß_1_, we next performed experiments using the sGC inhibitor ODQ prior to IRI and enucleated the eyes after 24 hours. We found that ODQ alone had no influence on sGC phosphorylation following IRI. However, ODQ reduced ALF-186 mediated protein expression of sGC in Western Blot. This effect was confirmed by densitometrical analysis where ODQ abolished the ALF-186 mediated increase of sGCß_1_-protein expression completely (Fig. 2c, d). Figure 3a shows the upregulation of sGC in the inner retina of ALF-186 treated eyes after IRI. Co-expression with Brn-3a confirmed the expression of sGC in RGCs. Quantification of sGC revealed a significant increase due to ALF-186 treatment, especially during IRI, while iALF-186 attenuated sGC signal (Fig. 3b; *** = *p* < 0.001).

### ALF-186 reduces IRI-induced increase of NF-κB mRNA expression and protein phosphorylation

**Fig. 4:**
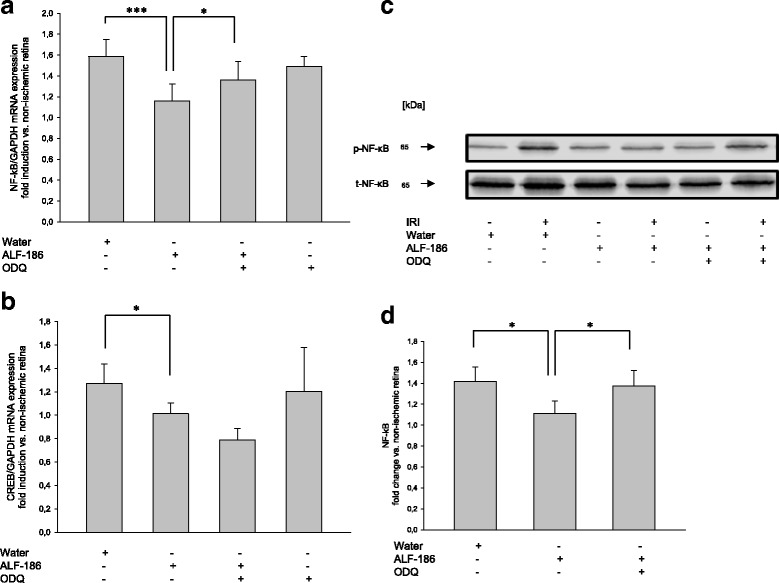
Effect of ALF-186 treatment on transcription factors NF-κB and CREB. **a** Fold induction of NF-κB mRNA expression after ALF-186 and sGC inhibition in ischemic retinal tissue compared to GAPDH in relation to the corresponding non-ischemic retinae analyzed by RT-PCR (*n* = 8; data are mean ± SD; IRI vs. IRI + ALF-186, *** = *p* < 0.001 and IRI + ALF-186 vs. IRI + ODQ + ALF-186, * = *p* < 0.05). **b** Fold induction of CREB mRNA expression in ischemic retinal tissue compared to GAPDH in relation to the corresponding non-ischemic retinae analyzed by RT-PCR (*n* = 8; data are mean ± SD; IRI vs. IRI + ODQ + ALF-186 * = *p* < 0.05). **c** Representative Western Blot image (*n* = 8) showing the suppression of phosphorylated NF-κB compared to total NF-κB after immediate ALF-186 treatment. Enucleation was performed 24 h post IRI. **d** Densitometric analysis of *n* = 8 Western Blots for phosphorylated NF-κB after ALF-186 and ODQ inhibition (data are mean ± SD; IRI vs. IRI + ALF-186, * = *p* < 0.05, IRI + ALF-186 vs. IRI + ODQ + ALF-186, * = *p* < 0.05)

Next, we have examined the influence of ALF-186 on transcription factors NF-κB and CREB by RT-PCR and Western Blot analysis. ALF-186 reduced IRI-mediated increase of NF-κB mRNA expression as well as NF-κB protein phosphorylation significantly (Fig. 4a; *** = *p* < 0.001; * = *p* < 0.05). At least in part, this effect can be repealed by sGC inhibition with ODQ (Fig. 4a; * = *p* < 0.05; Fig. 4c, d; * = *p* < 0.05).

Additionally, ALF-186 reduces IRI-induced increase of CREB mRNA, but not in a significant way. Surprisingly, pretreatment with ODQ before IRI and treatment with ALF-186 decreased CREB mRNA expression while ODQ had no effect at all (Fig. 4b).

### ALF-186 reduced IRI-induced increase of tumor necrosis factor alpha (TNF-α)

**Fig. 5:**
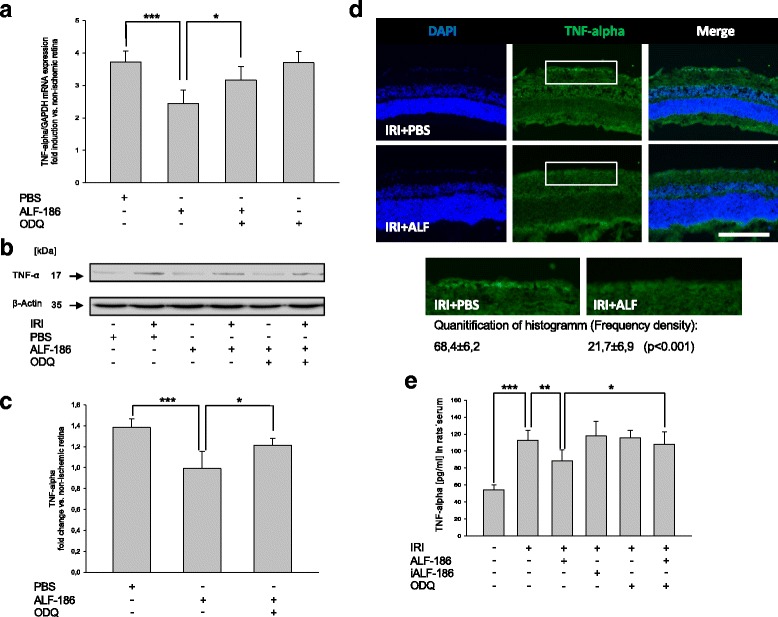
Effect of ALF-186 treatment on retinal protein and mRNA expression of TNF-α in tissue and TNF-α serum levels. **a** Fold induction of TNF-α mRNA expression after ALF-186 in ischemic retinal tissue compared to GAPDH in relation to the corresponding non-ischemic retinae analyzed by RT-PCR (*n* = 8; data are mean ± SD; IRI vs. IRI + ALF-186, *** = *p* < 0.001 and IRI + ALF-186 vs. IRI + ALF-186 + ODQ, * = *p* < 0.05). **b** + **c** Representative Western Blot image and densitometric analysis of *n* = 8 Western Blots showing the suppression of retinal cleavage of TNF-α compared to β-Actin after ALF-186 (data are mean ± SD; IRI vs. IRI + ALF-186, *** = *p* < 0.001) and it’s clearing after ODQ treatment (IRI + ALF-186 vs. IRI + ALF-186 + ODQ, * = *p* < 0.05). **d** Histological images, showing that TNF-α protein expression is increased due to IRI + PBS, while ALF-186 treatment attenuates this effect. Quantification via frequency density of TNF-α protein expression in both groups by histogram analysis revealed a significant downregulation of TNF-α due to ALF-186 treatment (*p* < 0.001). **e** TNF-α serum levels in peripheral blood serum after ALF-186 treatment (data are mean ± SD; *n* = 8; Sham vs. IRI, *** = *p* < 0.001; IRI vs. IRI + ALF-186, ** = *p* < 0.01 and IRI + ALF-186 vs. ODQ + IRI + ALF186, * = *p* < 0.05)

TNF-α is a cytokine that has been identified as a key regulator in inflammatory response. Therefore, we analyzed TNF-α mRNA and protein expression by RT-PCR and Western Blot. IRI increased TNF-α mRNA substantially. This effect was partially antagonized by sGC-inhibition (Fig. 5a; *** = *p* < 0.001; * = *p* < 0.05). Western Blot analysis confirmed this effect: ALF-186 decreased IRI induced increase of TNF-α protein expression while ODQ was able to reverse expression partly (Fig. 5b, c; *** = *p* < 0.001; ** = *p* < 0.01 and * = *p* < 0.05). TNF-α was detectable in retinal cross sections of IRI-eyes mainly in the area of the ganglion cell layer. Quantification of TNF-α revealed a significant decrease due to ALF-186 treatment during IRI. (Fig. 5d; *p* < 0.001). Serum levels of TNF-α protein revealed a substantial increase due to IRI (Fig. 5e; *** = *p* < 0.001), while the administration of ALF-186 significantly decreased TNF-α protein levels in the rats’ serum (Fig. 5e; ** = *p* < 0.01). Inhibition of sGC via ODQ inhibited the ALF-186 mediated effect regarding TNF-α serum protein expression (Fig. 5e; * = *p* < 0.05).

### ALF-186 reduced IRI-induced increase of Interleukin-6 (IL-6)

**Fig. 6:**
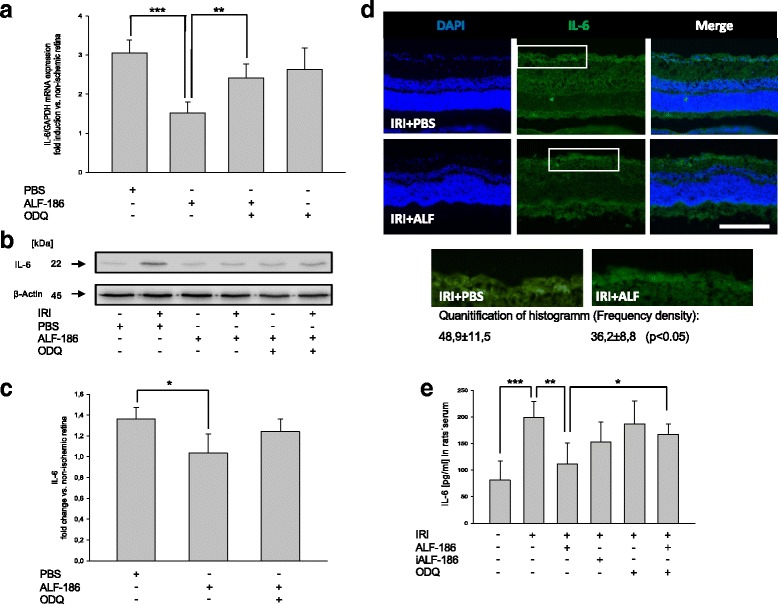
Effect of ALF-186 treatment on retinal protein and mRNA expression of IL-6 in tissue and IL-6 serum levels. **a** Fold induction of IL-6 mRNA expression in ischemic retinal tissue compared to GAPDH in relation to the corresponding non-ischemic retinae analyzed by RT-PCR (*n* = 8; data are mean ± SD; IRI vs. IRI + ALF-186, *** = *p* < 0.001, IRI + ALF-186 vs. IRI + ODQ + ALF-186, ** = *p* < 0.01). **b, c** Representative Western Blot image and densitometric analysis of *n* = 8 Western Blots showing the suppression of retinal IL-6 protein compared to β-Actin after ALF-186 (data are mean ± SD; IRI vs. IRI + ALF-186, * = *p* < 0.05). **d** Histological images, showing that IL-6 protein expression is increased due to IRI + PBS, while ALF-186 treatment attenuates this effect. Quantification via frequency density of IL-6 protein expression in both groups by histogram analysis revealed a significant downregulation of IL-6 due to ALF-186 treatment (*p* < 0.05). **e** IL-6 serum levels in peripheral blood serum after ALF-186 treatment (data are mean ± SD; *n* = 8; Sham vs. IRI, *** = *p* < 0.001; IRI vs. IRI + ALF-186, ** = *p* < 0.01 and IRI + ALF-186 vs. ODQ + IRI + ALF186, * = *p* < 0.05)

In the next step, we examined the influence of ALF-186 on the pro-inflammatory cytokine IL-6. ALF-186 caused a rigorous drop of IRI induced IL-6 mRNA expression (Fig. 6a; *** = *p* < 0.001). However, in a weakened form, this effect was also observed in IL-6 protein expression (Fig. 6b + c; * = *p* < 0.05). sGC inhibition diminished the ALF-186 mediated IL-6 mRNA decrease (Fig. 6a; ** = *p* < 0.01). IL-6 expression was predominantly present in the ganglion cell layer of retinal cross sections and slightly pronounced in IRI + PBS-eyes compared to ALF-treated eyes (Fig. 6d). Quantification of IL-6 revealed a significant decrease due to ALF-186 treatment during IRI (Fig. 6d; *p* < 0.05). Serum levels of IL-6 protein revealed a substantial increase due to IRI (Fig. 6e; *** = *p* < 0.001), while the administration of ALF-186 significantly decreased TNF-α protein levels in the rats’ serum (Fig. 6e; ** = *p* < 0.01). Inhibition of sGC via ODQ inhibited the ALF-186 mediated effect regarding TNF-α serum protein expression (Fig. 6e; * = *p* < 0.05).

### ALF-186 attenuates heat shock response

**Fig. 7:**
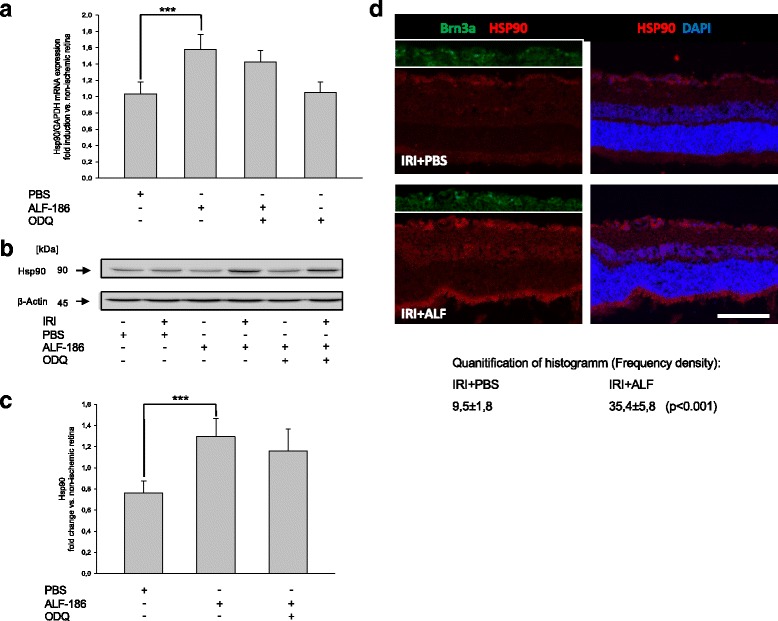
Effect of ALF-186 treatment on retinal expression of Hsp90 mRNA and protein. **a** Fold induction of Hsp90 mRNA expression in ischemic retinal tissue compared to GAPDH in relation to the corresponding non-ischemic retinae analyzed by RT-PCR after ALF-186 and sGC inhibition (IRI vs. IRI + ALF-186, *** = *p* < 0.001; *n* = 8; data are mean ± SD). **b**, **c** Representative Western Blot image and densitometric analysis of *n* = 8 Western Blots showing the increase of retinal Hsp90 protein compared to β-Actin after ALF-186 (data are mean ± SD; IRI vs. IRI + ALF-186, *** = *p* < 0.001). This effect was not counteracted by ODQ. **d** Compared to IRI + PBS, Hsp90 protein expression is increased by ALF-186 treatment, mainly in RGC and bipolar cells. Quantification via frequency density of Hsp90 protein expression in both groups by histogram analysis revealed a significant increase of Hsp90 due to ALF-186 treatment (*p* < 0.001)

**Fig. 8:**
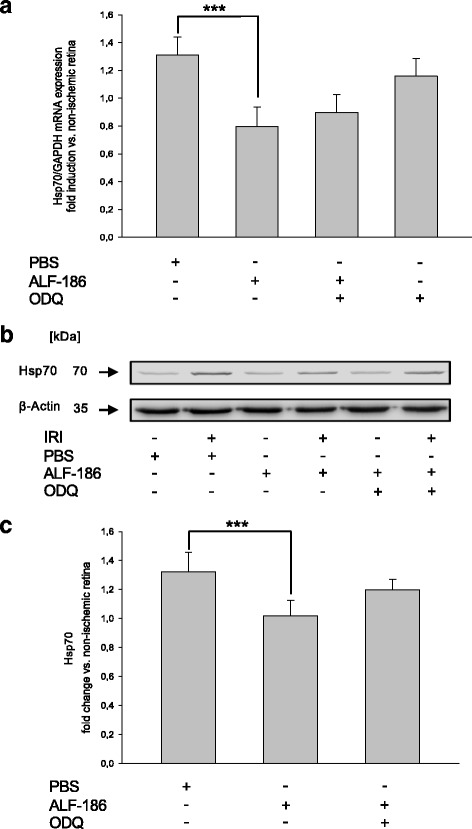
Effect of ALF-186 on retinal expression of Hsp70 mRNA and protein. **a** Fold induction of Hsp70 mRNA expression in ischemic retinal tissue compared to GAPDH in relation to the corresponding non-ischemic retinae analyzed by RT-PCR after ALF-186 and sGC inhibition (IRI vs. IRI + ALF-186, *** = *p* < 0.001; n = 8; data are mean ± SD). **b**, **c** Representative Western Blot image and densitometric analysis of *n* = 8 Western Blots showing the decrease of retinal Hsp70 protein compared to β-Actin after ALF-186 (data are mean ± SD; IRI vs. IRI + ALF-186, *** = *p* < 0.001)

In a further step, we analyzed the impact of IRI and ALF-186 on heat shock proteins 70 and 90. ALF-186 increased HSP90 mRNA and protein expression (Fig. 7a; *** = *p* < 0.001; Fig. 7b, c; * = *p* < 0.05). HSP-90 was expressed predominantly in RGC of IRI + ALF-treated eyes, as shown by double-immunohistochemistry with Brn-3a (Fig. 7d). In contrast, HSP70 mRNA and protein expression was reduced significantly by ALF-186 (Fig. 8a; *** = *p* < 0.001; Fig. 8b, c; * = *p* < 0.05).

Interestingly, inhibition of sGC did not alter heat shock response at all, neither in mRNA nor in protein expression.

## Discussion

The main findings of this in vivo study can be summarized as follows: (1) ALF-186 mediates neuroprotective properties via the soluble guanylate cyclase-β_1_ subunit (sGC-β_1_) in RGC. (2) ALF-186 diminished the IRI induced NF-κB expression and phosphorylation in RGC. (3) ALF-186 treatment reduced expression of inflammatory cytokines Interleukin-6 (IL-6) and tumor necrosis factor alpha (TNF-α) in neuronal tissue and in rats’ serum. (4) ALF-186 modified IRI-mediated heat shock response. (5) Inhibition of sGC-β_1_ abrogated ALF’s effects regarding NF-κB, IL-6 and TNF-α expression, finally leading to a decrease of retinal ganglion cells. (6) However, ODQ did not affect the heat shock response significantly.

Various studies have shown that low-dose carbon monoxide—given either before or after injury—may decrease organic failure: As a potential therapeutic agent, it has impact on different organic systems and mediates protection in lung, liver, kidney and cardiovascular system [17–19]. Well known as a potentially toxic agent at high doses, little is known about the protective mechanism of low-dose CO affecting neuronal tissue, especially not when given as a therapeutic and non-inhalational agent. Our data show that sGC-β_1_ seems to play a pivotal role in promoting ALFs’ neuroprotective effects. Moreover, other cellular processes seemed to be involved concerning ALF’s protective effect. Own research demonstrated that MAPK p38 is decisively involved in ALFs’ action on neuronal apoptosis and neuroprotection. Vieira et al. demonstrated in their study with primary cultured neurons that pre-treatment with CO exerted protection implicating sGC, NO and mitochondrial K_ATP_ channels [20]. Moreover, in a recently published work, Vieira et al. demonstrated the effectiveness of the CORM A1 in improving neurogenesis and preventing neuronal apoptosis [21]. Several studies have shown that carbon monoxide exerts protective effects due to IRI in different organic tissues by activation of sGC [2, 22, 23].

Furthermore, Wang et al. exposed mice to CO immediately after middle cerebral artery occlusion and detected reduced brain damage and proved Nrf2 pathway as a crucial part of the mechanism [24]. Another aspect was explored by Mahan et al. who claim changes in neurometabolic substrate, especially lactate, responsible for CO’s neuroprotective effect [25].

Regarding neuronal injury, the impact of carbon monoxide on pro-inflammatory NF-κB has not been investigated so far. However, Wei et al. attribute carbon monoxide hepato-protective effects in part to NF-κB. In their investigation with hepatic IRI in rats, CORM-2 inhibited the activity of NF-κB leading to decreased serum levels of pro-inflammatory cytokines IL-6 and TNF-α [26]. Qin et al. demonstrated the impact of CORM on the inflammatory response in a model of septic mice. CORM administration was able to inhibit sepsis-induced NF-κB activation in lung and liver and reduced serum cytokine levels of IL-6 and TNF-α [5]. For the first time, we describe the influence of CO on NF-κB phosphorylation and pro-inflammatory cytokine expression in neuronal tissue. According to the study of hepatic IRI we found a down-regulation of NF-κB and, consequently, IL-6 and TNF-α following ALF-186 treatment not only in neuronal tissue but in the serum, too, demonstrating the systemic effects of ALF-186.

The impact of carbon monoxide on neuronal inflammation has not been well analyzed. Our results showed a reduction of IRI induced IL-6 and TNF-α expression. These finding are in accordance with Biermann et al. who described similar effect on TNF-α when CO was inhaled prior to retinal IRI [16]. However, the anti-inflammatory effect of carbon monoxide due to IRI in non-neuronal tissue and organs is well described in literature. Nakao et al. explored in their transplant study not only jejunal circular muscle contractility after intestinal transplantation in rats, but also expression of inflammatory cytokines in the graft muscularis. They demonstrated that carbon monoxide improved muscle dysmobility and also decreased transplant induced IL-1β and IL-6 upregulation [2]. Neto et al. performed orthotopic kidney transplation in rats and demonstrated reduced mRNA levels of pro-inflammatory cytokines IL-1ß, IL-6, and TNF-α in kidney grafts of CO treated recipients [4]. Moreover, inhalative CO treatment may affect these cytokines in heart and lung grafts [3, 6].

In the present study, we also have examined the impact of ALF-186 regarding the heat shock response. The heat shock response is characterized by the cellular expression of heat shock proteins in response to harmful events like ischemia, heat or toxins. Heat shock proteins are described to weaken harmful stressors and play a crucial role in neuronal cytoprotection, affecting cell death and immune response pathways [27, 28]. Various studies indicate that Hsp-70 mediates anti-inflammatory effects and decreases inflammatory cytokine production. For example, increased levels of Hsp-70 are detected in cerebral IRI [29, 30]. Neuroprotective effects are described for Hsp-90 as well [31, 32]. However, the interactions and interdependencies between the Hsps are not well studied, especially not in neuronal inflammation. However, upon dissociation from their common transcription factor HSF, Hsp-90 is able to induce Hsp-70 indicating opposite effects [33]. Our data show that ALF-186 increased Hsp-90 expression after retinal IRI and, interestingly, a decrease in the expression of Hsp-70. This result are partly in contrariety to the findings by Biermann et al., showing an induction of Hsp-70 when inhaled carbon monoxide was applied prior to retinal IRI [16]. The mode and time-point of application may be responsible for these divergent results. It is tempting to speculate that ALF-186 is able to suppress cytokine production and provide robust protection inhibiting Hsp-70 and consecutively increasing Hsp-90.

Furthermore, we demonstrated that the specific sGC inhibitor ODQ was able to abrogate the effect of ALF-186 on transcription factor NF-κB as well as pro-inflammatory cytokines IL-6 and TNF-α, while no significant changes were observed concerning the heat shock response. Finally, ODQ mitigated ALF’s neuroprotective effect leading to an increased loss of retinal ganglion cells. This result is in accordance to Nakao et al. They demonstrate that sGC inhibitor ODQ was able to abrogate the beneficial effect of carbon monoxide during cold intestinal ischemia reperfusion injury associated with intestinal transplantation in rats. In accordance to our study ODQ also inhibited transplant-associated increase of pro-inflammatory IL-6 [2].

It is very interesting that neither ALF-186 nor IRI alone are able to increase retinal sGC expression, although the combination of both clearly increases sGC. We speculate about a “second hit” phenomenon, in which both parts are needed to induce sGC expression. ODQ preferentially binds to the prosthetic heme part of sGC, thus inhibiting both, activity and subsequently its de-novo expression. As known throughout the literature, sGC activation may alter vascular smooth muscle cell proliferation, platelet aggregation and consequently blood flow [15, 34]. We cannot exclude, that ALF-186 additionally activates smooth vascular muscle sGC in the retina. This would potentially lead to an increase of retinal blood flow due to sGC-mediated vasodilation and could alter the reperfusion injury [35].

Our model has some limitations: The rats’ retina is a very small organ and even though desirable, it is not possible to perform many assays with one retina. In order not to kill to many animals for just one experiment, we chose to perform nRNA and protein expression assays, rather than activity assays, although activity—especially of sGC—would elucidate the role of ALF-186 in the context of IRI even more.

## Conclusion

In conclusion, the carbon monoxide-releasing molecule ALF-186 was able to reduce IRI mediated inflammation, by upregulating sGC-β1, as well as suppressing the pro-inflammatory transcription factor NF-κB and CREB and its downstream cytokines IL-6 and TNF-α in neuronal tissue and systemically in the rats’ serum. Furthermore, ALF-186 differentially regulated the heat shock response, thus proposing a novel target in neuronal IRI.

## Abbreviations

AMC: Activated microglia
CO: Carbon monoxide
CORM: Carbon monoxide releasing molecule
CREB: cAMP responsive element binding protein
DMSO: Dimethylsulfoxid
FG: Fluorogold
GCL: Ganglion cell layer
HSP: Heat shock protein
IL-6: Interleukin 6
MAPK: Mitogen activated protein kinase
NF-κB: Nuclear factor κB
ODQ: sGC inhibitor
PBS: Phosphate buffered saline
RGC: Retinal ganglion cell
sGC: Soluble guanylate cyclase, IRI, ischemia-reperfusion injury
TNF-α: Tumor necrosis factor-α
